# Consumption of 100% Orange Juice in Relation to Flavonoid Intakes and Diet Quality Among US Children and Adults: Analyses of NHANES 2013–16 Data

**DOI:** 10.3389/fnut.2020.00063

**Published:** 2020-05-13

**Authors:** Matthieu Maillot, Florent Vieux, Colin Rehm, Adam Drewnowski

**Affiliations:** ^1^MS-Nutrition, Marseille, France; ^2^Montefiore Medical Center, Albert Einstein College of Medicine, New York, NY, United States; ^3^Center for Public Health Nutrition, University of Washington, Seattle, WA, United States

**Keywords:** orange juice, socio-demographic, HEI-2015, Nutrient Rich Food (NRF) index, NHANES, flavonoid

## Abstract

This study explored consumption patterns of 100% orange juice by socio-demographics among US children and adults. Dietary intakes data for 15,983 persons aged >2 y came from the nationally representative National Health and Nutrition Examination Survey (NHANES 2013–2016). The What We Eat in America nutrient composition database was merged with the USDA Expanded Flavonoid Database to assess flavonoid intakes. Diet quality measures were the Healthy Eating Index (HEI-2015) and Nutrient Rich Food (NRF9.3) Index. Orange juice consumption accounted for a mean of 14 kcal/d and varied with age, incomes, and race/ethnicity. Orange juice consumption was associated with higher intakes of bioactive flavonoids, lower added sugars, and higher-quality diets overall. Diets of consumers were higher in vitamin C, potassium, calcium, vitamin D (adults), flavanones, and total flavonoids (children) as compared to non-consumers. Consumers had significantly higher HEI-2015 and NRF9.3 scores and lower body mass index values (adults). However, only 15.9% of the NHANES sample consumed any orange juice at all; of these 11.8% had <1 serving/day and only 3.4% had 1 serving/day or more.

## Introduction

The consumption of 100% fruit juice has been associated with increased risk of childhood obesity (1, 2). Dietary Guidelines for Americans recommend the consumption of whole fruit as opposed to 100% juice on the grounds that juice “can contribute extra calories when consumed in excess” (3). Yet the consumption of 100% fruit juices in the US is relatively low; sugar-sweetened beverages are consumed far more often and in greater amounts, especially by older children and young adults (4). In past studies, beverage patterns built around milk and 100% juice were associated with higher-quality diets than were beverage patterns built around SSB (4).

Orange juice and apple juice are the two principal 100% fruit juices in the US diet (5). Whereas the consumption of sugar-sweetened (6, 7) and other beverages (4) is well-described, fewer analyses have explored orange juice consumption patterns. This project used two cycles of NHANES data (2013–2016 NHANES) to explore orange juice consumption by socio-demographics among US children and adults. One novel feature was the use of the USDA expanded flavonoid database (8) in dietary intake assessment. Citrus juices are an important source of bioactive flavanols in the US diet (9).

The present hypothesis was that orange juice consumption would be associated with higher flavonoid intakes. Insofar as orange juice can replace sugar sweetened beverages, we also expected to see higher-quality diets among consumers of orange juice as opposed to non-consumers.

## Materials and Methods

Dietary intakes data for 15,983 persons (5,919 children 2–19 y and 10,064 adults aged >20 y) came from 1 or 2 days of the National Health and Nutrition Examination Survey (2013–2016 NHANES). In the NHANES computer-aided 24-h recall, participants were asked to report the types and amounts of all food and beverages consumed during the prior 24-h, from midnight to midnight. The first recall identified a quick list of foods and beverages consumed, along with time and occasion for each food item. A second pass was then conducted to record amounts consumed and was followed by a final probe for any frequently forgotten foods. Day 1 interviews were conducted in a mobile examination center by trained dietary interviewers. For children 2–5 y, dietary recall was completed by a parent or guardian who had knowledge of the child's diet. For children 6–11 y, dietary recall was aided by a proxy respondent who was also present. Children 12–19 y were the primary source of dietary recalls but could be assisted by an adult who had knowledge of their diet.

### Participant Characteristics

The NHANES sample was stratified by gender (male, female) and by age. The age categories were toddlers (2–3 y); young children (4–8 y); older children (9–13 y); adolescents (14–19 y), and adults. Adults were stratified into 20–30 y, 31–50 y, 51–70 y, and >70 y age groups. These age groups generally correspond to the age groups used by the US National Academies (10). The cut-points for the family income-to-poverty ratio (IPR) were: <1; 1–1.99; 2–3.49; and ≥3.5 (11). Demographic NHANES questionnaires were used to stratify the sample by race/ethnicity, defined here as non-Hispanic white; non-Hispanic black, Mexican American, other Hispanic, other/mixed race. The level of education for adults was defined as: < high school, high school, some college, college graduate, or higher. Weight status was measured using BMI (body mass index kg/m2) and waist circumferences. Weight status of children was measured using BMI *z*-scores split into four classes using clinically meaningful cut-points. The ethics board review for the NHANES data collection is documented by the National Center for Health Statistics online (12). Analyses of federal NHANES data are exempt from approvals by Institutional Review Boards.

### Classification of Beverages

Beverages were classified into six categories: 1. Orange juice 100%; 2. Other 100% citrus juices; 3. Non-citrus juices, including apple and 100% vegetable juices; 4. Milk and milk beverages; 5. Drinking water (tap and bottled); 6. Other caloric beverages (>50 kcal/8 oz); 7. Other low calorie beverages (<50 kcal/8 oz); 8. Baby formula. The 100% fruit juice blends (e.g., apple-cranberry) were included in the 100% juice category but sweetened fruit-based drinks with added sugars were placed among other caloric or low-calorie beverages based on their energy density. Baby formula was included, although the consumption was low. Breast milk was consumed by very few toddlers > 2 y.

The USDA Food Data Central (13) lists the nutrient composition of 100 g of freshly squeezed 100% orange juice as 45 kcal, 8.4 g total sugars (0 g added sugars), 50 mg vitamin C, 200 mg of potassium, 11 mg calcium, and 0.2 g dietary fiber.

### Measures of Diet Quality

Energy and nutrient intakes from 24-h dietary recalls were calculated using the Food and Nutrient Database for Dietary Studies (FNDDS 2011–2014), customized with the addition of vitamin D and added sugars. These nutrient composition data were supplemented with data from the Food Patterns Equivalents Database (FPED) from the United States Department of Agriculture (USDA) (14). The FNDDS database was further merged with the USDA Expanded Flavonoid Database released in 2016 (15). The Flavonoid Database contains analytical values for 29 flavonoids as well as class totals. The unit of measure was mg/100 g edible portion on fresh weight basis. Flavonoid classes were flavan-3-ols, flavanones, flavonols, anthocyanidins, flavones, and isoflavones, Among the flavanones were hesperetin, naringenin, and eriodictyol.

The HEI-2015 was designed by the USDA to monitor compliance with the 2015 Dietary Guidelines for Americans (16). The HEI-2015 is a 100-point scale that assesses adequate consumption of fruits (10 points), vegetables (10), grains (10), dairy (10), protein foods (10), and fats (10). The HEI-2015 food adequacy items are based around food groups to encourage, with some food categories specified by name. Those include total vegetables, dark-green and orange vegetables, total fruit, whole fruit, whole grains, total protein foods, protein from seafood and plant sources, and total dairy. The foods to moderate or limit were refined grains, sodium, added sugars, and saturated fats.

The Nutrient Rich Foods (NRF9.3) index was a second measure of dietary nutrient density (17–19). It was based on nine nutrients to encourage (NR9 subscore) and three nutrients to limit (LIM subscore). Reference daily values (DVs) followed the US Food and Drug Administration values and other standards (20). The nine nutrients to encourage were: protein (50 g), fiber (28 g), vitamin A (900 RAE), vitamin C (90 mg), vitamin D (20 mcg), calcium (1,300 mg), iron (18 mg), potassium (4,700 mg), and magnesium (420 mg). The maximum recommended values (MRVs) for nutrients to limit were: added sugar (50 g), saturated fat (20 g), and sodium (2,300 mg). The NRF9.3 was calculated as follows:

$$\begin{array}{l} NRF\;9.3d=\left(NR-LIM\right)x\;100, \end{array}$$

where

$$\begin{array}{lll} NR & = & \overset{9}{\underset{i=1}{\sum }}\frac{\frac{Intake_{i}}{Energy}\times 2000,}{DV_{i}}\\ LIM & = & \overset{3}{\underset{i=1}{\sum }}\frac{\frac{Intake_{i}}{Energy}\times 2000,}{MRV_{i}}-1. \end{array}$$

The NRF family of nutrient density indices is well-documented (21). In recent versions, vitamin E was replaced with vitamin D, a nutrient of public health concern (3). Both NRF9.3d and HEI 2015 were corrected for dietary energy (1,000 kcal for HEI-2015 and 2,000 kcal for NRF) (16). Percent DVs were truncated at 100% following prior protocols. In LIM, only the MRV share in excess of the recommended amount was considered.

### Plan of Analysis

The percentage of orange juice consumers and mean amounts consumed (in g/day and kcal/day) were estimated by gender, age group, IPR (income-to-poverty ratio), and race/ethnicity. The contribution of orange juice to daily energy and nutrient intakes were estimated for the whole sample and by age group and other strata of interest. Orange juice consumers and non-consumers were compared on nutrient intakes, HEI-2015 and NRF9.3d, and on weight status using BMI for adults and BMI *z*-scores for children/adolescents. All analyses accounted for the complex survey design of NHANES data and are representative of the US population. Data analyses used Stata 13.1 (College Station, TX) and SAS 9.4 (SAS institute, Cary, NC).

## Results

Table 1 shows participant characteristics for persons aged >2 y in NHANES 2013–16. The sample was 61.8 % Non-Hispanic White; 11.9% Non-Hispanic Black, 10.8% Mexican American, 6.4% other Hispanic, 9.1% other, and was evenly distributed by gender and by income-to-poverty ratio (IPR).

**Table 1:** Consumption of 100% orange juice (OJ) as % consumers and amounts consumed in grams/d and kcal/d by socio-demographic variables.

	***N***	**%**	**OJ % consuming**	**OJ (g/d)**	**OJ (kcal/d)**
Overall	15,983	100	15.9 (14.8–16.9)	29.8 (27.6–31.9)	14.2 (13.2–15.3)
**Gender**					
Female	8,172	51.05	14.3 (13.2–15.5)	23.0 (21.2–24.8)	11.0 (10.1–11.8)
Male	7,811	48.95	17.5 (16.0–19.0)	36.8 (33.2–40.5)	17.6 (15.9–19.4)
*p*-value			0.0009	<0.0001	<0.0001
**Age**					
2–3 y	723	2.64	18.1 (14.3–21.9)	28.5 (20.0–37.1)	13.7 (9.5–17.9)
4–8 y	1,685	6.50	21.2 (17.4–25.1)	29.0 (22.2–35.7)	13.9 (10.6–17.1)
9–13 y	1,658	6.53	18.6 (15.2–22.1)	30.5 (24.4–36.7)	14.7 (11.7–17.6)
14–19 y	1,853	8.44	15.5 (12.9–18.1)	31.5 (25.8–37.2)	15.2 (12.4–17.9)
20–30 y	1,875	15.87	15.4 (13.2–17.5)	36.4 (29.4–43.5)	17.5 (14.1–20.9)
31–50 y	3,453	25.96	13.1 (11.7–14.5)	28.2 (24.5–32.0)	13.5 (11.7–15.3)
51–70 y	3,314	25.26	14.0 (11.9–16.0)	23.4 (20.2–26.6)	11.1 (9.6–12.6)
>70 y	1,422	8.81	24.2 (20.5–27.8)	39.2 (34.4–44.0)	18.6 (16.4–20.9)
*p*-value			<0.0001	0.0001	0.0001
**Poverty level**					
<1	3,819	16.17	17.9 (15.7–20.1)	37.3 (31.4–43.2)	17.8 (15.0–20.7)
1–1.99	3,950	20.39	17.6 (15.1–20)	31.6 (26.1–37.1)	15.1 (12.4–17.7)
2–3.49	3,105	21.16	14.0 (11.8–16.3)	27.2 (22.6–31.8)	12.9 (10.8–15.1)
>3.49	3,841	35.57	15.0 (13.2–16.8)	26.1 (22.1–30)	12.5 (10.6–14.4)
Missing	1,268	6.71	16.1 (12.7–19.6)	34.0 (25.1–42.9)	16.3 (12.0–20.6)
*p*-value			0.06	0.04	0.04
**Race ethnicity**					
Non-Hispanic White	5,586	61.81	13.8 (12.5–15.1)	24.3 (21.9–26.7)	11.6 (10.4–12.7)
Non-Hispanic Black	3,476	11.93	21.5 (20–23.1)	41 (37.1–44.9)	19.7 (17.8–21.6)
Mexican American	2,865	10.75	19.5 (16.7–22.2)	42.1 (34.3–49.9)	20.2 (16.4–23.9)
Other Hispanic	1,789	6.36	20.9 (18.1–23.7)	41.5 (34.1–48.9)	19.8 (16.3–23.3)
Other/mixed race	2,267	9.14	14.7 (12.3–17.1)	29.5 (23.6–35.4)	14.1 (11.3–17.0)
*p*-value			<0.0001	<0.0001	<0.0001

*The data are means and confidence intervals (95% CI)*.

Table 1 shows percent consumers in each group and the amounts consumed in g/d and kcal/d. On average, only one person in six consumed orange juice (15.9%). Males were more frequent consumers compared to females (17.5 vs. 14.3%). Percentage of consumers declined from 21.2% in the 4–8 age group to a low of 14% in the 51–70 y age group but rose to 24.2 after age 70 y. Most likely to consume orange juice were non-Hispanic Blacks and other Hispanics; least likely were non-Hispanic Whites.

Mean consumption of orange juice was <1 ounce per person per day (29.8 g/d). Males consumed more orange juice than did females (36.8 vs. 23.0 g/d). Consumption first rose and then fell with age, reaching a peak for the >70 y group (39.2 g/d). Total energy intakes from orange juice followed the same patterns. Orange juice contributed from 11.1 to 18.6 kcal/d, depending on age. Orange juice intakes declined with rising incomes. Whereas Mexican Americans derived as much as 20 kcal/d from orange juice, non-Hispanic Whites were at 11 kcal/d.

Table 2 shows the contribution of orange juice and other beverages to daily energy and nutrient intakes. Orange juice and other citrus juices contributed 14.4 kcal/d on the average; other fruit juices (mostly apple juice) contributed 14.8 kcal/d. By contrast, milk contributed 75.5 kcal/d and sugar sweetened caloric beverages 212.5 kcal/d. Orange juice and other citrus juices contributed a mean of 2.5 g/d of total sugar and 0 tbs/d of added sugar. High calorie beverages contributed 31.8 g/d of total sugar and 7 tbs/d (i.e., 28 g/d) of added sugar. Mean daily energy contribution from orange juice was only 0.7% of the total. Orange juice contributed 13.2% of daily vitamin C and about 2% each of potassium and calcium. By contrast, high calorie beverages contributed 10.5% of total energy, 29.9% of total sugars, and 43.7% of added sugars, and had less favorable nutrient-to-energy ratios.

**Table 2:** The contribution of OJ and other beverages to energy and nutrient intakes in the US population aged >2 y.

**Beverage category**	**Energy, kcal/d**	**Fiber, g/d**	**Vitamin C, mg/d**	**Calcium, mg/d**	**Potassium, mg/d**	**Total sugar, g/d**	**Added sugar, tsp/d**
Orange juice	14.2 (13.2–15.3)	0.1 (0.1–0.1)	10 (10-11)	20 (18-22)	53 (49–57)	3 (2–3)	0 (0–0)
Other citrus juice	0.2 (0.1–0.3)	0 (0–0)	0 (0–0)	0 (0–1)	1 (0–1)	0 (0–0)	0 (0–0)
Non-citrus juice	14.8 (13.5–16.2)	0.1 (0.1–0.1)	7 (7–8)	5 (5–6)	38 (35-42)	0 (0–1)	0 (0–0)
Milk	75.5 (71.5–79.4)	0.1 (0.1–0.1)	0 (0–0)	178 (169–188)	208 (196–220)	8 (8–9)	0 (0–0)
Other caloric	212.5 (201.5–223.5)	0.3 (0.3–0.3)	9 (9-10)	45 (41–49)	152 (142–161)	32 (30-34)	7 (7–8)
Other non-caloric	6.4 (5.9–6.8)	0 (0–0)	2 (2)	10 (9-11)	138 (127–148)	0 (0–0)	0 (0–0)
Water	0.4 (0.3–0.5)	0 (0–0)	0 (0–0)	62 (59–65)	0 (0–1)	0 (0–0)	0 (0–0)
Formula	0.3 (0.2–0.5)	0 (0–0)	0 (0–0)	0 (0–1)	0 (0–1)	0 (0–0)	0 (0–0)
Beverages total	324.3 (313.0–335.7)	1 (1)	29 (28–31)	321 (311–330)	590 (573–606)	44 (42–45)	7 (7–8)
Diet total	2,016 (1,990–2,043)	16.4 (16.0–16.8)	77 (75–80)	965 (943–988)	2,512 (2,467–2,556)	106 (104–108)	16 (16–17)

*Data are means and 95% CI. Added sugars are shown in teaspoons (1 tsp = 4 g)*.

Table 3 shows percentage of individuals consuming 0 servings, <1 serving, 1–1.99 servings, or at least two servings per day of orange juice. The serving is 8 oz as per FDA RACC, the data were separated for children and adults. Only 18% of children and 15% of adults consumed any orange juice at all. Of those 14.73% of children and 10.91% of adults consumed one serving per day or less. Only 2.91% of children and 3.6% of adults consumed between one and two servings. About one in 200 children (0.54%) and one in 160 adults consumed two servings of orange juice or more per day. On average, persons consuming >2/d servings of orange juice consumed 661 g/d, equivalent to 264 kcal/d.

**Table 3:** Average orange juice consumptions and percentage of individuals consuming 0 servings, <1 serving, 1–1.99 servings, or at least two servings per day of orange juice.

	**Overall**	**0 servings/d**	** <1 serving/d**	**1–1.99 servings/d**	**≥2 servings/d**
**Overall**					
% Consumers	100	84.13	11.83	3.43	0.61
OJ intakes, g/d	29.8 (27.6–31.9)	0 (0–0)	125.2 (122.0–128.3)	318.9 (311.4–326.3)	661.3 (613.9–708.7)
**Children**					
% Consumers	100	81.83	14.73	2.91	0.54
OJ intakes, g/d	30.2 (26.2–34.2)	0 (0–0)	117.3 (112.9–121.7)	326.9 (314.0–339.9)	635.2 (582.0–688.4)
**Adults**					
% Consumers	100	84.86	10.91	3.60	0.63
OJ intakes, g/d	29.6 (27.4–31.8)	0 (0–0)	128.5 (124.9–132.1)	316.8 (307.9–325.7)	668.5 (608.8–728.1)

Table 4 shows diet quality indicators for orange juice consumers and non-consumers. Population means were adjusted for energy intake, gender, poverty level, race ethnicity, and education level (for adults only). For children, orange juice consumption (on the NHANES days) was associated with significantly higher vitamin C, potassium, calcium (*p* < 0.0001), and vitamin D (*p* < 0.0004). Although total sugar was higher, added sugar was significantly lower (*p* = 0.003). Despite fears that fruit juice is lower in fiber than whole fruit, orange juice consumption was not associated with lower intakes of dietary fiber—if anything the trend was in the opposite direction.

**Table 4:** Diet quality indicators for consumers and non-consumers of OJ.

	**Children**			**Adults**		
	**Non-consumers**	**Consumers**	***P***	**Non-consumers**	**Consumers**	***P***
*N* (%)	4,689 (81.8)	1,230 (18.2)		8,433 (84.9)	1,631 (15.1)	
Fiber, g/d	14 (13.7–14.3)	14.6 (14.0–15.1)	0.09	17.4 (17.0–17.8)	17.7 (17.0–18.4)	0.42
Vitamin A RE, mcg/d	583.8 (567.8–599.8)	572.6 (539.9–605.2)	0.49	613.0 (592.1–634.0)	630.6 (591.0–670.3)	0.39
Vitamin C, mg/d	65.1 (62.2–68.0)	118.6 (113.2–124.0)	<0.0001	73.5 (70.0–77.0)	139.7 (134.6–144.8)	<0.0001
Vitamin D, mcg/d	5.4 (5.2–5.6)	5.5 (5.1–5.8)	0.67	4.5 (4.3–4.7)	5.3 (4.9–5.6)	<0.005
Calcium, mg/d	951.2 (933.1–969.3)	1,015.5 (978.7–1,052.3)	<0.005	876.7 (861.6–891.9)	989.3 (961.5–1,017.2)	<0.0001
Potassium, mg/d	2,084.9 (2,058.2–2,111.6)	2,309.9 (2,262.4–2,357.3)	<0.0001	2,538.1 (2,503.6–2,572.6)	2,790.1 (2,739.0–2,841.2)	<0.0001
**Total flavonoids**						
mg per d	77 (65.1–88.9)	108.6 (94.7–122.4)	<0.005	188.5 (173.9–203.0)	191.9 (156.0–227.8)	0.86
mg per 2,000 kcal	89.8 (77.0–102.7)	122.3 (109.2–135.5)	<0.0001	203.8 (189.6–218.0)	204.6 (169.7–239.5)	0.97
**Total flavanones**						
mg per d	5.1 (4.4–5.9)	36.8 (34.6–38.9)	<0.0001	6.1 (5.2–7.0)	44.1 (42.0–46.1)	<0.0001
mg per 2,000 kcal	6.3 (5.3–7.3)	41.8 (39.0–44.6)	<0.0001	7.5 (6.1–8.8)	46.9 (44.1–49.6)	<0.0001
Total sugar, g/d	104.3 (102.2–106.5)	107.7 (104.2–111.3)	0.16	101.7 (100.0–103.4)	110.7 (107.7–113.7)	<0.0001
Added sugar, g/d	15.1 (14.5–15.6)	13.5 (12.6–14.3)	<0.01	15.7 (15.2–16.2)	13.8 (13.1–14.6)	<0.0001
HEI 2015	48.5 (47.8–49.1)	51.6 (50.3–52.9)	<0.0001	51.3 (50.6–51.9)	54.7 (53.9–55.4)	<0.0001
NRF9.3	427.9 (422.7–433.1)	466.6 (458.1–475.1)	<0.0001	419.5 (412.7–426.2)	475.4 (466.5–484.3)	<0.0001
NR9, %/2,000 kcal	5.7 (5.7–5.8)	6.0 (6.0–6.1)	<0.0001	5.5 (5.5–5.6)	6.1 (6.0–6.1)	<0.0001
LIM, %/2,000 kcal	1.4 (1.4–1.4)	1.4 (1.4–1.4)	<0.005	1.3 (1.3–1.4)	1.3 (1.3–1.3)	<0.005

*Data are means adjusted for energy intake, gender, poverty level, race ethnicity, and education level (for adults only) and 95% CIs. Data is from NHANES 2013–16*.

For adults, orange juice consumption (on the NHANES days) was associated with significantly higher vitamin C, potassium, and calcium (*p* < 0.0001) and significantly lower consumption of added sugar (*p* = 0.003). Orange juice consumption was not associated with lower intakes of dietary fiber.

Orange juice consumption among children was associated with significantly higher intakes of flavanones and of total flavonoids. Flavanones made an important contribution to total flavonoid intakes in children. Adult consumers had significantly higher intakes of flavanones. The increase in total flavonoids was not significant, partly because adults obtain flavonoids from other dietary sources such as tea.

Healthy Eating Index HEI 2015 scores were significantly higher for orange juice consumers than for non-consumers. The effect was significant for children and for adults. The NRF9.3 nutrient density scores were significantly higher for orange juice consumers than for non-consumers. The effect was significant for children and for adults. The NR9 nutrient density score was higher for consumers. The effect was significant for children and for adults. The LIM subscore was significantly lower for orange juice consumers as compared to non-consumers, even though the difference was small.

Table 5 shows adjusted averages of BMI for adults and BMI *z*-scores for children for orange juice consumers and non-consumers. Also shown are waist circumferences among children and adults by orange juice consumption status. Adjustment was for total energy, gender, race-ethnicity, poverty level, and education level (for adults only). No differences in BMI *z*-scores or waist circumferences were observed for children. Adult consumers of orange juice had lower BMI values and lower waist circumferences than did non-consumers.

**Table 5:** Comparison of adjusted averages of anthropometric measures between consumers and non-consumers of OJ among children and adults, 2013–2016.

	**Children**			**Adults**		
	**Non-consumers**	**Consumers**	***P***	**Non-consumers**	**Consumers**	***P***
*n*	4,689	1,230		8,433	1,631	
%	81.83	18.17		84.86	15.14	
BMI, *z*-score	0.63 (0.57–0.69)	0.6 (0.48–0.73)	0.63			
BMI, kg/m^2^				29.48 (29.21–29.75)	28.74 (28.16–29.33)	<0.05
Waist circumference	69.64 (69.03–70.25)	69.28 (68.19–70.38)	0.52	99.91 (99.22–100.6)	97.87 (96.49–99.25)	<0.005

*Adjustment was for total energy, gender, race-ethnicity, poverty level, and education level (for adult only)*.

## Discussion

Based on a representative sample of 15,983 US toddlers, children, adolescents, and adults, the present analyses show that the average consumption of 100% orange juice was much lower than it is commonly assumed. Only 15.8% of the NHANES sample consumed any orange juice on the NHANES data collection days. Of these, 11.8% had one serving/day or less and only 0.61% of the sample had two servings/day or more. Orange juice contributed only around 0.7% of total energy and no added sugars to the diets. These low levels of consumption were well within the current national guidelines of up to 4–8 oz. total fruit juice per day. Orange juice and apple juice are the two principal 100% juices in the US diet.

Orange juice consumers had diets with more dietary vitamin C, potassium, calcium, and vitamin D for adults. Both HEI-2015 and NRF9.3d scores were significantly higher among orange juice consumers as compared to non-consumers. Up to five points on the HEI 2015 score can be earned through the consumption of 0.8 cups of orange juice per 1,000 kcal. Similarly, since vitamin C, vitamin D, calcium, and potassium are among the components of the NRF9.3 score, orange juice consumption ought to be associated with higher scores. There is also the possibility, grounded in prior research (22), that consuming orange juice is a marker of healthier eating habits generally. Foods are consumed as part of food patterns and not in isolation. In past studies based on 1999–2008 data, consumers of low-calorie sweeteners had better HEI 2005 subscores for vegetables, whole grains, and low fat dairy, even though diet beverages lack any of those components (23). Consumers of low calorie sweeteners were less likely to smoke and more likely to engage in physical activity (23). Similarly, healthier diets associated with the consumption of orange juice may have benefits that go beyond the nutrient content of orange juice itself.

The current recommendations for adopting plant-based diets rest in part on the health benefits of plant-derived flavonoids and other bioactive compounds (24). In the US diet, most total flavonoids and nearly all flavan-3-ols are provided by fermented black tea; flavan-3-ols from tea and anthocyanidins from berries have been associated with lower risk of cardiovascular diseases (25, 26). Children do not drink tea (11) but drink fruit juice. Even though the consumption of orange juice was low, significant differences in flavanol intakes were observed between orange juice consumers and non-consumers. The difference was significant for both children and adults. Children who consumed orange juice also had significantly higher intakes of total flavonoids.

The role of 100% fruit juice in US children's diets continues to be a topic of debate (2, 27–30). The position taken by the American Academy of Pediatrics is that 100% fruit juice “predisposes to excessive caloric intake” and “has no essential role in healthy, balanced diets of children” (2). Same as any other food, “juice can contribute extra calories when consumed in excess” (2). However, statements that “children 2–18 years of age consume nearly half of their fruit intake as juice” (2) are not consistent with current evidence. First, we have previously shown that whole fruit accounted for 66% of total fruit consumption; there was no indication that 100% juice displaced whole fruit in any way (31). Other studies have noted that the reported decline in 100% juice consumption was not accompanied by a corresponding increase in servings of whole fruit (32). Rather, 100% juice may improve diet quality of lower SES groups by removing the added costs associated with whole fruit consumption (33). Total fruit consumption among lower-income participants in the Special Supplemental Nutrition Program for Women, Infants, and Children (WIC) was higher than among non-participants and came mostly from 100% fruit juice rather than whole fruit (34). Juice was the cost neutral way to meet the fruit shortfall for lower income groups (33).

Despite concerns that the consumption of juice may lower dietary fiber intakes (2), there was no difference in fiber consumption between consumers and non-consumers of orange juice. Despite concerns that 100% juice consumption by children is associated with higher risk of childhood obesity, there were no significant differences in BMI *z*-scores or waist circumferences between child consumers and non-consumers of orange juice. For adults, orange juice consumption was associated with lower BMI and lower waist circumferences, adjusting for multiple covariates. However, given the cross-sectional design of NHANES studies, no causal inferences can be made.

Other studies have described the evidence to link 100% fruit juice intake with overweight status or adiposity as insufficient (27). Past analyses of NHANES data (4, 35) showed no association between 100% juice consumption and BMI *z*-scores. A recent meta-analysis (5) found that 100% fruit juice led to small, not clinically significant, weight gain in children 1–6 y and no weight gain in older children (7–18 y). The type of juice was noted as a potential reason: younger children drink apple juice, whereas older children are more likely to drink citrus juice (5). There was no consistent evidence to suggest that 100% fruit juice was associated with increased body weight or key metabolic risk factors (35, 36).

The 2015–20 Dietary Guidelines have taken a more nuanced view of 100% juice consumption, one that stresses the concept of nutrient density, generally expressed as the ratio of nutrients to calories. First, there was the recognition that beverages such as “milk and fruit and vegetable juices contain important nutrients such as calcium, potassium, and vitamin D in addition to calories” (3). Those nutrients were indeed higher in diets of consumers as opposed to non-consumers of orange juice. The 2015–2020 DGA went on to say that “beverages that are calorie-free—especially water—or that contribute beneficial nutrients, such as fat-free and low-fat milk and 100% juice, should be the primary beverages consumed” (3). In other words, what counted in the DGA was the nutrient-to-calorie ratio, the basic principle behind the present nutrient density scores. The DGA position that beverage drinking patterns built around milk, 100% juice, and water were associated with higher quality diets were also confirmed in past research (4). However, that study also showed that such patterns were relatively rare; the consumption of sugar sweetened beverages was the norm.

The present study had limitations. The cross-sectional nature NHANES data does not permit the drawing of causal associations. The present discussion was therefore limited to associations. Dietary intakes data were self-reported and included proxy reports by caregivers for children 5 years or younger. Despite these shortcomings, NHANES data are still used as the basis for dietary policies in the US. Nutrient profiling models such as the NRF9.3 that are purely nutrient based may not adequately capture multiple aspects of healthy food patterns. The same limitation applies to the Healthy Eating Index 2015, developed by the USDA to measure compliance with the 2015 USDA dietary guidelines. The HEI-2015 may not adequately capture food patterns across diverse social strata.

## Conclusions

In both children and adults, the consumption of orange juice was associated with higher quality diets and lower intakes of added sugar. No deficits in fiber intake were observed. Orange juice consumption was not associated with overweight. Orange juice consumers had higher intakes of dietary flavanones and total flavonoids (children).

## Data Availability Statement

The datasets analyzed for this study can be found in the CDC National Center for Health Statistics NHANES database at: https://wwwn.cdc.gov/nchs/nhanes/Default.aspx.

## Ethics Statement

The studies involving human participants were reviewed and approved by National Center for Health Statistics. Written informed consent to participate in this study was provided by the participants' legal guardian/next of kin.

## Author Contributions

MM, FV, CR, and AD designed the study. CR developed the databases. MM and FV conducted the analyses. AD took the lead on writing the paper. All authors read and approved the final manuscript.

## Conflict of Interest

AD has received grants, contracts, and honoraria from numerous entities, both public and private, for studies on dietary nutrient density and nutrient profiling of individual foods and food patterns. FV and MM are employed by MS-Nutrition, a consulting firm. The remaining author declares that the research was conducted in the absence of any commercial or financial relationships that could be construed as a potential conflict of interest.
